# Food Safety Risk Information-Seeking Intention of WeChat Users in China

**DOI:** 10.3390/ijerph17072376

**Published:** 2020-03-31

**Authors:** Zhaohui Yang, Krishna P. Paudel, Xiaowei Wen, Sangluo Sun, Yong Wang

**Affiliations:** 1 College of Economics and Management, South China Agricultural University, Guangzhou 510642, China; zhaohuiyang@stu.scau.edu.cn (Z.Y.); sunsangluo@stu.scau.edu.cn (S.S.); wzyongs@126.com (Y.W.); 2 Department of Agricultural Economics and Agribusiness, Louisiana State University (LSU) and LSU Agricultural Center, Baton Rouge, LA 70803, USA; kpaudel@agcenter.lsu.edu

**Keywords:** food safety, Risk Information Seeking and Processing model, risk perception, WeChat user

## Abstract

Consumers’ food safety risk information-seeking behavior plays a vital role in improving their food quality and safety awareness and preventing food safety risks. Based on the Risk Information Seeking and Processing Model (RISP), this paper empirically analyzes the food safety risk information-seeking intention of consumers in WeChat and influencing factors under the impact of food safety incidents. We use data from 774 WeChat users and apply the Structural Equation Modeling (SEM) approach. We also conduct multigroup analysis with demographic characteristics as moderating variables. The results demonstrated that: (1) Risk perception (*p* ≤ 0.01) has direct significant positive effects on consumers’ intention to seek food safety information. Besides, higher risk perception (*p* ≤ 0.01) regarding food safety risks will make people feel more anxious and threatened, and then expand the gap between the information they need and the relevant knowledge they actually have (*p* ≤ 0.1), which will further stimulate them to seek more information (*p* ≤ 0.05). (2) Informational subjective norms (*p* ≤ 0.01) can not only directly affect consumers’ information-seeking about food safety, but also indirectly affect consumers’ intention through information insufficiency (*p* ≤ 0.01). (3) The more consumers trust the relevant channels (*p* ≤ 0.01), the stronger their intention to search for food safety risk information. Moreover, the multiple-group analysis also shows that the effects of consumers’ gender, age, educational background, and average monthly earnings are different among different groups. Furthermore, implications are put forward for food safety risk communication efforts in China.

## 1. Introduction

Since the reform and opening up in 1978, China’s economy has developed rapidly, people consume a variety of food items, and they are using more meat and dairy products. At the same time, especially after China’s accession to the World Trade Organization (WTO) in 2001, there was a sharp increase in food safety risks and many food safety incidents in the country [1]. The data obtained by a big data-mining firm show the mainstream network media reported about 408,000 food safety incidents in China from 2008 to 2017 with an average of 112 food safety incidents per day [2]. One of the important reasons for the outbreak of a series of food safety incidents in China is the shortcoming of food safety laws, regulations, and supervision systems [3,4]. From 2003 to 2012, China implemented a multisectoral segmented food safety supervision, which seriously reduced the efficiency of food safety supervision [1]. Food Safety Law of the People’s Republic of China implemented in 2009 did not fundamentally improve the flaws in China’s food safety supervision system. Food safety incidents not only restrict the healthy development of China’s food industry, damaging the international image of China, but also attack people’s confidence in food safety [3]. To improve food safety, the Chinese government has implemented a series of policies and measures, such as reforming relevant laws, strengthening major surveillance, and monitoring systems [5]. After 2013, a unified food safety supervision system was formed after a new round of food safety supervision mechanism reform. In October 2015, the newly revised Food Safety Law of the People’s Republic of China, known as the most stringent food safety law in Chinese history was officially promulgated and implemented [4]. The food safety strategy has risen to become one of the major national strategies (website: http://www.gov.cn/zhengce/2019-05/20/content_5393212.htm). The overall situation of food safety in China has been improving in recent years. However, compared with the United States, the European Union, and other developed countries or regions, China’s food safety regulatory system still needs to be further improved. Food safety concerns in China are still severe, and China is still facing threats and challenges from many food safety risk factors [3]. Food safety is still one of the extremely serious issues of public concern in China and the public perceive lots of food safety risk and anxieties [6].

Consumers are the final link in the food supply chain, their attention, and understanding of food safety information can promote producers and retailers to pay more attention to the control of food quality and safety, which is also an indispensable part in food safety governance. Therefore, the research on consumers’ food safety risk information-seeking behavior is crucial. Information asymmetry is the root cause of the food safety problem [7,8,9,10]. The quality and safety of food have the property of the trust. Although consumers cannot completely identify the quality and safety of the product, they could minimize the degree of information asymmetry by searching for food quality and safety information. In the research of risk-faced decision-making and behavior, Griffin et al. proposed the Risk Information Seeking and Processing (RISP) model based on the Heuristic-Systematic Model (HSM) of information processing and Theory of Planned Behavior (TPB) [11]. The RISP model has been applied in many fields [12], such as to understand people’s antinuclear behavioral intentions [13], risk of drinking tap water drawn from the lakes [14], risk of flooding, global warming or climate change [15,16,17], disease health-related risk [18,19], and food-related risk [20]. The general adaptability of the RISP model has been widely verified. Food safety risks are diverse and are very closely related to people’s life. There is a limited study related to food safety risk based on the RISP model. Additionally, there are limited studies to validate Chinese consumers’ risk-seeking behavior related to food safety, especially the risk-seeking behavior of the consumers based on their characteristics. In addition, WeChat is one of the most important social media platforms in China, and its number of monthly active users has soared from 0.36 billion in 2013 to 1.15 billion as of June 2019, ranking third in the world after WhatsApp and Facebook. According to the WeChat Influences Report 2018, the information consumption driven by WeChat reached RMB 209.7 billion, accounting for 4.7% of China’s total information consumption, and it also accounts for 34% of China’s mobile traffic consumption.

Under such a circumstance, this paper analyzes the food safety risk information-seeking intention and the influencing factors of consumers based on the RISP model and Multigroup Structural Equation Modeling (SEM) analysis by using WeChat users’ questionnaire survey. Our goal is to understand the impact of different demographic characteristics on consumers’ behavior. The application of the RISP model to the research of consumers’ risk-seeking behavior in food safety is of considerable significance not only to promote its theoretical use in the field of food safety but also to improve the ability of the consumers to overcome food safety risks.

## 2. Theoretical Framework and Hypotheses

**Figure 1:**
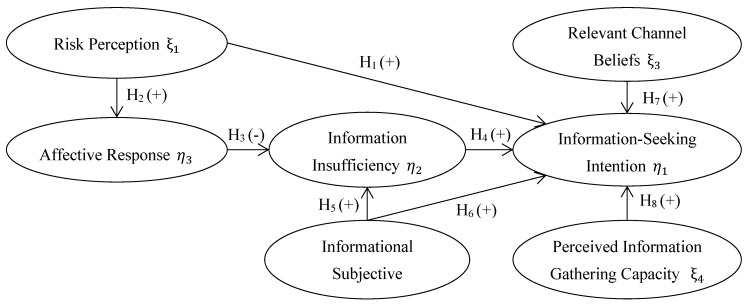
RISP model framework and research hypotheses. Notes: $\eta_{1}$,$\;\eta_{2}$,$\;\mathrm{and}\;\eta_{3}$ are latent variables as outcomes; $\xi_{1}$,$\;\xi_{2}$,$\;\xi_{3}$,$\;\mathrm{and}\;\xi_{4}$ are latent variables as causes; H_1_–H_8_ are tested hypotheses with ‘+’ sign inside parentheses indicating a positive relationship between two latent variables and ‘−’ sign inside parentheses indicating a negative relationship between two latent variables.

Individual characteristics, risk perception, affective response, information insufficiency, informational subject norms, relevant channel beliefs, and perceived information gathering capacity affect an individual’s risk information collection process [11]. These are the components in the RISP model, which are shown in Figure 1. In the succeeding paragraphs, we outline these components and their role in the risk information collection process.

### 2.1. Risk Perception

Risk refers to the possibility and consequences of adverse events [21,22], which is related to the degree of future losses [23]. From the perspective of risk theory, the risk related to food safety is defined as a function of the probability of an adverse health effect and the severity of that effect, consequential to a hazard(s) in food [24]. Risk perception is also perceived hazard characteristics, which is used to describe people’s cognitive evaluations of potential risk [15]. High risk perception may increase the uncertainty of consumers, so that they will obtain more information to access the risk, thus stimulating the consumers to search for more information [25,26]. If consumers judge that food safety risk will threaten their personal health or their families’ health, they may have a stronger willingness to search for more food safety information in order to cope with risks. In addition, affective response is placed after risk perception in the RISP model, which implies an affective response is always caused by risk perception. Affective response that accompanies the risky situation is essentially negative [15]. Consumers with higher risk perception may react more negatively to food safety incidents. Therefore, the following hypotheses are advanced:

**Hypothesis 1** **(H1).** *Consumers with higher risk perception have a higher information-seeking intention about food safety risk*.

**Hypothesis 2** **(H2).** *Consumers with higher risk perception have a more negative affective response to food safety incidents*.

### 2.2. Affective Response

Affective response refers to emotional reactions to risks [27]. In the RISP model, this kind of emotional reaction could affect people’s judgment of the amount of risk information they feel needed, more specifically, it may influence people’s sense of information sufficiency toward risk [11,16]. Food safety is closely related to consumers’ life and health. Food safety incidents that have occurred were the external manifestation of objective food safety risks [28]. In the face of food safety incidents, the affective response, such as worry, anxiety, etc., could prompt the consumers to generate a sense of the inadequacy of quality and safety information. It in turn generates the intention of food safety information so as to take effective action to deal with food safety risks. Accordingly, we forward the following hypothesis:

**Hypothesis 3** **(H3).** *Affective response to risk increases consumers’ information search for food safety*.

### 2.3. Information Insufficiency

Information insufficiency is the gap between the knowledge held and the information needed of consumers, which is a motivational factor of risk information-seeking behavior in the RISP model [11]. This kind of size gap will ultimately influence individuals’ information seeking and processing styles [29]. The positive relationship between information insufficiency and information-seeking behavior in different risks contexts has been confirmed by many papers [17,18]. We propose the following hypothesis:

**Hypothesis 4** **(H4).** *Information insufficiency has a positive effect on consumers’ food safety risk information-seeking intention*.

### 2.4. Informational Subjective Norms

One of the basic theories of the RISP model is the theory of planned behavior (TPB). Therefore, the concept of informational subjective norms mainly comes from the subjective norm of the TPB. Subjective norm is the person’s perception of social pressures (in other words, the salient individuals or groups) put on him to perform or not perform the particular behavior. If a consumer believes he should perform a certain behavior based on what the society wants he is likely to perform it [30,31]. Subsequently, informational subjective norms emphasize a normative influence from the social pressures they perceived in the process of risk information collection, this influence can mobilize an individual desire for information sufficiency [32]. There are two meanings of information subjective norm: one is the expectation that society wants consumers to know particular risk information; the other is the enthusiasm that consumers comply with society’s expectations. Only when consumers have enough enthusiasm to defer to the expectation of knowing a certain risk that the society has placed on them, can the informational subjective norms really play a role in our seeking and processing behavior of risk information [32]. Thus, the hypotheses are as follows:

**Hypothesis 5** **(H5).** *Informational subjective norms positively influence consumers’ information insufficiency about food safety risk*.

**Hypothesis 6** **(H6).** *Informational subjective norms positively influence consumers’ food safety risk information-seeking intention*.

### 2.5. Relevant Channel Beliefs

Relevant channel beliefs refer to the trusting attitude of consumers to the direct government risk control department, universities, and news media that provide risk-related information. This belief mainly includes the perception of the reliability and validity of the risk information released by the above relevant channels, which could affect consumers’ information seeking and processing strategies they may employ [33]. Relevant channel beliefs could influence consumers’ choices for risk information seeking and processing strategies. We proposed the following hypothesis:

**Hypothesis 7** **(H7).** *Relevant channel beliefs of consumers have a positive effect on consumers’ food safety risk information-seeking intention*.

### 2.6. Perceived Information Gathering Capacity

Perceived information gathering capacity measures the level of an individual’s ability to collect relevant risk information. Generally speaking, when consumers are faced with food safety risks and need to inquire relevant information and knowledge, the fewer obstacles they face, the easier it is for them to query the information, and the higher intention to search relevant risk information they will have. Consequently, the following hypothesis is formulated:

**Hypothesis 8** **(H8).** *Perceived information gathering capacity of consumers has a positive effect on their food safety risk information-seeking intention*.

## 3. Materials and Methods

### 3.1. Methodology

A typical Structural Equation Modeling (SEM) not only can express the relationship between the latent variables and their indicators but also describe the relationship between endogenous and exogenous variables and the relationship among the endogenous variables [34]. Therefore, we chose the SEM as the main method and the equations used are as follows:

(1) $$\eta_{1}=\beta_{12}\eta_{2}+\gamma_{11}\xi_{1}+\gamma_{12}\xi_{2}+\gamma_{13}\xi_{3}+\gamma_{14}\xi_{4}+\zeta_{1}\;\eta_{2}=\beta_{23}\eta_{3}+\gamma_{22}\xi_{2}+\zeta_{2}\;\eta_{3}=\gamma_{31}\xi_{1}+\zeta_{3}$$

There are seven latent variables that cannot be observed directly. Information-Seeking Intention (ISI), Information Insufficiency (II), and Affective Response (AR) are the latent variables as outcomes, i.e., exogenous variables, which are represented by $\eta_{1}$, $\eta_{2}$, and $\eta_{3}$. Risk Perception (RP), Informational Subjective Norms (ISN), Relevant Channel Beliefs (RCB), and Perceived Information Gathering Capacity (PIGC) are the latent variables as causes, i.e., endogenous variables, which are denoted by $\xi_{1}$, $\xi_{2}$, $\xi_{3}$, and $\xi_{4}$. In Equation (1), $\beta_{12}$ indicates the degree of influence of information insufficiency ($\eta_{2}$) on the information-seeking intention ($\eta_{1}$). $\gamma_{11}$, $\gamma_{12}$, $\gamma_{13}$, and $\gamma_{14}$ respectively represent the degree of influence of the exogenous variables $\xi_{1}$, $\xi_{2}$, $\xi_{3}$, and $\xi_{4}$ on the information-seeking intention ($\eta_{1}$). $\beta_{23}$ indicates the influence of affective response ($\eta_{3}$) to information insufficiency ($\eta_{2}$). $\gamma_{22}$ denotes the influence of informational subjective norms ($\xi_{2}$) on information insufficiency ($\eta_{2}$) and $\gamma_{31}$ indicates the influence of risk perception ($\xi_{1}$) on the affective response ($\eta_{3}$). Finally, $\zeta_{1}$, $\zeta_{2}$, and $\zeta_{3}$ are the residual terms of the regression equations.

### 3.2. Data Collection

Questionnaires were sent to consumers on WeChat between June and August 2017. Convenience sampling was used in this research and it is in accordance with the sampling methods adopted in many similar papers conducted using social media survey [35,36,37]. Sojump (https://www.wjx.cn/) is a powerful humanized questionnaire survey platform in China, which can provide a professional online questionnaire survey. After developing the questionnaire on the web page through the Sojump, we linked it into WeChat, a well-known social media platform in China, and collected 803 responses. After eliminating 29 invalid responses, there were 774 valid responses left. The reason for choosing WeChat is that WeChat is more reliable than other social media platforms. In China, WeChat Pay and Alipay are the two main electronic payment methods with very high popularity. True personal information of every WeChat user who uses WeChat Pay needs to be certificated with a series of formal procedures from the WeChat based on China’s information regulation. Without real-name authentication, not only the payment function in WeChat cannot be used, but also some other functions will be restricted. Hence, we have faith in that the respondents of the study are who they say they are. The sample size meets the requirement of structural equation model analyses (generally speaking, 200 or more observations are required to conduct SEM based analyses [38]). China has a population of about 1.43 billion as of 2019, while WeChat has 1.11 billion monthly active users. Excluding the elderly people and children, the most majority of Chinese consumers use WeChat.

## 4. Results

### 4.1. Descriptive Statistics of Variables and Demographic Characteristics of Consumers

On the basis of the previous papers using the RISP model, we used the Likert five-point scale as the measurement method and designed an appropriate scale as far as possible in order to guarantee the content validity of the questionnaire. All the measurement items were measured as “strongly disagree” (=1), “disagree” (=2), “feel neutral” (=3), “agree” (=4), and “strongly agree” (=5) depending on the respondents’ degree of agreement with the indicators, respectively. Scale design and descriptive statistics of all measurement items are displayed in Table 1.

Table 2 below shows the demographic characteristics of the sample. As for the gender ratio, 54.9% of the respondents were female and 45.1% were male. In terms of age, the proportion of respondents under 35 years was 65.5% and the remaining 34.5% were equal or older than 35 years. Respondents with a bachelor’s degree or higher occupied 78.9% of responses, while the respondents with below a bachelor’s degree accounted for 21.1%. There were 16% who did not report their average monthly earnings, excluding these samples, the proportion of average monthly earnings below or equal to 5000 CNY (around the US $750) is the same as that of average monthly earnings above 5000 CNY, which is 42%.

### 4.2. Reliability, Validity, and Confirmatory Factor Analysis

Before testing the research hypotheses, we conducted the reliability, validity, and confirmatory factor analysis by using statistical analysis software SPSS 22.0 and LISREL 8.7. The relevant results are reported in Table 3 and Table 4. As far as the reliability of the scale is concerned, the measurement indicators mainly include the Corrected Item-Total Correlation (CITC), Cronbach’s α value, and Composite Reliability (CR) [39,40]. In Table 3, the CITC of each measurement ranges from 0.670 to 0.893, which are higher than the cutoff value of 0.5, the Cronbach’s α of each latent variable ranges from 0.85 to 0.93, which greater than 0.7, and the CR of every latent variable ranges from 0.848 to 0.928, which is greater than 0.5. That means the scale of our research has high reliability. As for the validity, the Average Variance Extracted (AVE) in Table 3 is greater than the recommended value of 0.5, showing that the latent variables have good convergence validity. The square root of the AVE of each latent variable is larger than the correlation coefficients between one latent variable and other latent variables, implying that the latent variables have better discriminant validity (see Table 4). Kaiser–Meyer–Olkin (KMO) measure of sampling adequacy of all the latent variables was 0.908 and Bartlett’s test of sphericity was significant at 1%. According to the results of the confirmatory factor analysis, the standard factor loadings of each measurement item was greater than 0.7.

### 4.3. Structural Equation Model Analysis

#### 4.3.1. Offending Estimates Analysis

Offending estimates refer to the fact that the estimated coefficients output in the structural mode or the measurement mode exceeds the acceptable range. Generally, offending estimates occur in three ways: (1) there are negative or other meaningless error variances; (2) the standardization coefficients exceed or close to 1; and (3) the standard error is too big [40]. In the model, the error variances are between 0.10 and 0.43, and there are no negative or other meaningless error values. We also found that the standardization coefficients are much lower than 1, which indicates that there is no problem of offending estimates in the model. Thus, we can make further analysis.

#### 4.3.2. Model Fitting Test

Testing the fit effect is one of the most cardinal preceding steps in SEM analysis. Table 5 shows the overall fit evaluation results of the SEM model. There are three classifications of fit indices: absolute fit measures, incremental fit measures, and parsimonious fit measures [41]. As we can see from Table 5, except for a ratio of chi-square to the Degrees of Freedom (χ^2^/df) and Goodness of Fit Index (GFI), the actual values of the rest of fit indices were in line with a range of suggested values. As for χ^2^/df, its actual value was very close to the recommended values. This is because when using the indicators related to the chi-square distribution to test the fit effect of the model, it is sensitive to sample size [39]. Specifically, it means that it is easy to reject the model with a good fit effect when the sample size used is large [42]. The actual value of GFI was also very close to the suggested value. GFI ranges from 0 to 1, 0 represents the poor fit and 1 represents the perfect fit [43], and the GFI has a downward bias if the degree of freedom is large compared with the sample size (the degrees of freedom was 216 in our study). Due to the restrictiveness of the “sample-size” problem, the χ^2^/df and GFI actual values were acceptable. In general, the hypothetical model presented in this study fits well the actual observation data.

#### 4.3.3. Research Hypothesis Testing

**Figure 2:**
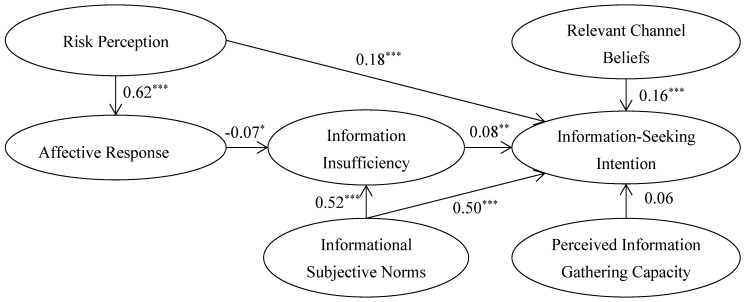
Path coefficients of Structural Equation Model. Notes: ***, **, and * indicated that *p*-values are significant at 1%, 5%, and 10% levels, respectively.

After the offending estimates and model fitting analysis, the research hypotheses between latent variables of the structural model were tested by using LISREL 8.7 software. The estimation results are presented in Table 6 and Figure 2. In general, except for the H8, the other seven hypotheses passed the test at different levels of significance.

The standardized coefficient of risk perception to consumers’ food safety risk information-seeking intention was 0.18 and was significant at 1% (H_1_; t = 4.86). It can be seen that the higher the consumers’ risk perception of food safety is, the stronger their attention to the search for food safety information. Risk perception also had a significant positive influence on the affective response, the path coefficient was 0.62 (H_2_; t = 17.32, *p* < 0.01), which means that the consumers with higher risk perception had a more negative affective response to food safety incidents. Therefore, we failed to reject H_1_ and H_2_. Next, the affective response had a negative influence on the information insufficiency and information insufficiency had a positive influence on information-seeking intention. The standardized path coefficients of H_3_ and H_4_ were −0.07 and 0.08, which were significant at a 10% level (H_3_; t = −1.73) and 5% (H_4_; t = 2.30), respectively. This implies consumers with a stronger negative emotional response to food safety incidents are more likely to have a sense of information insufficiency, and thus have a strong intention to seek food safety information. Hence, we failed to reject H_3_ and H_4_.

Informational subjective norms can indirectly affect food safety information-seeking intention of the consumers through information insufficiency. The path coefficient of informational subjective norms to information insufficiency was 0.52 (H_5_; t = 12.37, *p* < 0.01). Informational subjective norms also could directly influence information-seeking intention, and its standardized coefficient was 0.50 (H_6_; t = 9.26, *p* < 0.01). Results indicate that the social pressure from important individuals or groups were one of the most important factors affecting consumer’s information-seeking intention for food safety supporting hypotheses H_5_ and H_6_.

Relevant channel beliefs had a positive significant impact on consumers’ information-seeking intention related to food safety. We found the path coefficient to be 0.16 (H_7_; t = 4.18, *p* < 0.01). However, contrary to our belief, the influence of perceived information gathering capacity to information-seeking intention was not significant (H_8_; t = 1.60). Therefore, H_7_ was supported but H_8_ was not.

### 4.4. Multiple-Group Analysis

Taking the demographic characteristics of consumers as the moderator variables, the multiple-group analysis results of social media users’ food safety risk information-seeking intention are shown in Table 7. The analysis results of grouped samples and all the samples were generally similar.

In the path H_1_ where risk perception had a positive influence on the information-seeking attention, we could see that male (b = 0.23, *p* < 0.01) was more significant than female (b = 0.13, *p* < 0.05), the young group under 35 years old (b = 0.19, *p* < 0.01) was more significant than the older group over or equal to 35 years old (b = 0.13, *p* < 0.10). For the group with a bachelor’s degree or higher (b = 0.20, *p* < 0.01), the positive impact of risk perception on information-seeking intention was significant, while the group with below a bachelor’s degree was not significant. As for the average monthly earnings, compared with the group whose average monthly earnings was less than 5000 CNY (b = 0.10, *p* < 0.10), the positive influence of risk perception on information-seeking intention was more significant among the group whose average monthly earnings was more than 5000 CNY (b = 0.26, *p* < 0.01). This is because the male, young consumers, the group with a higher education degree and earnings usually have a higher risk-awareness, so their intention to seeking food safety information in order to reduce risk is generally higher.

In the path H_2_ where risk perception had a significant positive influence on affective response to food safety incidents, the multiple-group results demonstrated that although the impact of risk perception on affective response was more significant in female (b = 0.63, *p* < 0.01), young (b = 0.65, *p* < 0.01), and high-earning group (b = 0.66, *p* < 0.01), but the path coefficients gap was very small. In addition, the standardized coefficients of H_2_ between the group with a bachelor’s degree or higher and the group with below a bachelor’s degree were both 0.63 (*p* < 0.01). This indicates that no matter which group, when people are concerned about food safety risks, they will have normal affective response such as anger, annoyance, and worries about food safety incidents.

In the path H_3_ where affective response had a negative influence on the information insufficiency, it was manifested that the path coefficients between the young group and old group, low-earning and high-earning group did not pass the significance test. The male (b = −0.13, *p* < 0.05) and the group with below a bachelor’s degree (b = −0.20, *p* < 0.01) were significant while the female and the group with a bachelor’s degree or higher were not. Men are more rational and aware of the crisis in most cases, so their negative affective response to food safety incidents could quickly translate into a sense of lack of food safety information, which drives them to search for more information. As for the low-educated group, the relevant food safety knowledge may be insufficient. Therefore, they are likely to feel that they need more food safety information under the influence of the negative emotion to food safety incidents.

In the path H_4_ where information insufficiency had a positive influence information-seeking intention, we can see that female (b = 0.12, *p* < 0.05), young group (b = 0.09, *p* < 0.05), highly educated (b = 0.13, *p* < 0.01), and high-income group (b = 0.12, *p* < 0.05) were significant. This shows that when consumers think that their knowledge of food quality and safety is insufficient, the female, young group, highly educated, and high-income group are more likely to transfer this sense of information deficiency into the seeking intention of food safety.

In the path H_5_ where informational subjective norms had a positive influence on information insufficiency, all groups were significant at 1% statistical level, indicating that the pressure from the salient individuals or groups will give the consumers in different groups a sense of information insufficiency. Compared to the female (b = 0.40, *p* < 0.01), older group (b = 0.51, *p* < 0.01), highly educated (b = 0.43, *p* < 0.01), and high-income group (b = 0.26, *p* < 0.01), this effect was greater for male (b = 0.66, *p* < 0.01), young group (b = 0.54, *p* < 0.01), low-educated (b = 0.83, *p* < 0.01), and low-income consumers (b = 0.57, *p* < 0.01).

In the path H_6_ where informational subjective norms had a direct positive influence on information-seeking intention, the results in all the groups were significant, however, the effects were slightly different in different groups. Additionally, it was higher in male (b = 0.51, *p* < 0.01), older consumers (b = 0.63, *p* < 0.01), low-educated (b = 0.69, *p* < 0.01), and low-income consumers (b = 0.59, *p* < 0.01).

In the path H_7_ where relevant channel beliefs had a direct positive influence on information-seeking intention, the male (b = 0.21, *p* < 0.01) had a greater impact than female (b = 0.11, *p* < 0.05), the young consumers (b = 0.18, *p* < 0.01) had a greater impact than the older consumers (b = 0.13, *p* < 0.05), and the high-income consumers (b = 0.22, *p* < 0.01) had a greater impact than the low-income consumers (b = 0.11, *p* < 0.10). In addition, the highly educated consumers (b = 0.16, *p* < 0.01) had a significant effect but low-educated consumers did not.

Finally, the path H_8_ did not pass the significance test in the structural equation model, nevertheless, we found that in the multigroup analysis, the perceived information gathering capacity had a significant influence on seeking information intention among female (b = 0.10, *p* < 0.05), young (b = 0.14, *p* < 0.01), low-educated (b = 0.18, *p* < 0.05), and low-income consumers (b = 0.23, *p* < 0.01).

## 5. Discussion

We used RISP as a reference to construct a conceptual framework of consumers’ food safety risk information-seeking intention, and then assessed critical factors of consumers’ food safety risk information-seeking intention based on the responses from 774 WeChat users. The main conclusions from this research are as follows: (1) Risk perception is an important variable in predicting people’s information-seeking intention, consistent with previous studies [11,15,25]. On the one hand, risk perception has a direct significant positive effect on consumers’ information-seeking intention of food safety risk and this effect was more pronounced among the young, male, highly educated, and high-income groups. On the other hand, the higher risk perception regarding food safety risks will make people feel more anxious and threatened, and then expand the gap between the information they need and the relevant knowledge they actually have, which will further stimulate them to seek more information. (2) The social pressure that people perceived was also a significant determinant of their information-seeking intention, supporting those of previous studies [18]. Informational subjective norms can not only directly affect consumers’ information-seeking about food safety, but also indirectly affect consumers’ intention through information insufficiency. Moreover, male, low-education and low-income groups were more likely to feel this social pressure from the important others. (3) The more the consumers trust the relevant channels that publish the food safety-related information, the stronger their intention to seek food safety risk information, especially for male, young, highly educated, and high-income groups. The result was consistent with previous studies [27]. Our findings provide valuable implications for the efforts of food safety risk communication in China even in other countries. Firstly, the food safety information released to consumers can combine a certain degree of possible threats and potential consequences [44]. In this way, consumers’ risk perception and attention to food safety can be improved, so they could actively search for more information related to food safety and give full play to their role in food safety supervision. This is conducive to forcing food production enterprises to produce more safe and high-quality products, so as to improve the overall level of food safety in China. Secondly, reliable food safety information should be provided to consumers in accordance with the principles of science, accuracy, openness, and transparency, so as not to mislead consumers and public opinion. As for the government, a unified food safety information platform should be established to publish food safety risk warning information, investigation and handling information of major food safety accidents, and other important food safety information that need to be published. Besides, it is necessary to ensure that the information published is accurate and timely. As for news media, the report and publicity of food safety information should be true and fair. As for inspection organizations, the accuracy of the food safety inspection and testing reports they publish should be strictly guaranteed, and the contents in the report should be easy to understand and unambiguous. If necessary, the testing methods they applied should be clearly explained. Finally, strengthen food safety education for consumers and let them fully realize their role in food safety supervision.

## 6. Conclusions

In summary, our study further verified the applicability of the RISP theoretical model in the field of food safety in China. Furthermore, the effects of consumers’ characteristics such as gender, age, education, and income among different groups were analyzed. Compared with other literature, our research enriches the application of the RSIP model and also expands the depth of research by considering the impact of different demographic characteristics on consumers’ intention as previous studies did not empirically analyze the moderating effects of demographic variables. However, there are still the following deficiencies in our paper. (1) We did not expand the knowledge body of the RISP model. Therefore, future research could open up the new derivative model and research vision based on the RISP model or its derivative model, making its application more scientific and targeted. (2) The RISP model involved not only the content of information-seeking behavior but also the information processing behavior after information-seeking. This study did not analyze the risk information processing behavior, which is one of the limitations of this paper. So, the researchers could adopt more scientific indicators to measure the information processing behavior of consumers and strengthen the relevant research. (3) Other issues that may limit the interpretation of the results are that an online survey may have under-coverage and self-selection bias problems [45]. Many papers that used an online survey have pointed out those issues [46,47,48]. Limited by some facts, for example, WeChat is not open source, and there is no specific sampling frame, we can only implement convenient sampling, which may lead to biased estimates. Hence, it is necessary to alleviate these concerns in future research. Experimental, quasi-experimental, and field-experimental design may be considered to solve this problem.

## Figures and Tables

**Table 1:** Scale design and descriptive statistics.

Latent Variables	Measurement Items	Mean	S.D.
Risk Perception (RP)	RP1. Food safety issues have a real impact on my family and me.	4.01	0.890
	RP2. Food safety incidents seriously threaten my health.	3.93	0.940
	RP3. Food safety incidents seriously threaten my family’s health.	3.92	0.933
	RP4. Food safety issues seriously threaten the whole society.	4.13	0.863
Affective Response (AR)	AR1. The food safety incidents made me feel angry.	4.19	0.797
	AR2. The food safety incidents made me feel annoyed.	4.16	0.814
	AR3. The food safety incidents made me feel worried.	4.32	0.762
Information Insufficiency (II)	II1. I have more information about food safety risks currently.	3.04	0.930
	II2. I know what kinds of food safety risks I am facing.	3.18	0.931
	II3. I have enough knowledge in the face of food safety risks.	2.85	0.966
Informational Subjective Norms (ISN)	ISN1. People important to me think that I should stay at the top of information about food safety risk.	3.51	0.863
	ISN2. My family expects me to seek more information about food safety risk.	3.72	0.834
	ISN3. I think I should get more information about food safety risk.	3.97	0.805
Relevant Channel Beliefs (RCB)	RCB1. I trust the food safety information issued by the government.	3.62	0.875
	RCB2. I trust the food safety information issued by news media.	3.48	0.827
	RCB3. I trust the food safety information issued by researchinstitutions.	3.76	0.846
Perceived Information Gathering Capacity (PIGC)	PIGC1. If I want to find the information about food safety risk, I know where to find it.	3.38	0.915
	PIGC2. If I want to find the information about food safety risk, I know how to find it.	3.37	0.922
	PIGC3. I have already got the information I need related to food safety risk.	3.07	0.941
	PIGC4. It’s easy for me to obtain information about foodsafety risk.	3.02	0.988
Information-Seeking Intention (ISI)	ISI1. I intend to seek information related to food safety risk.	3.75	0.786
	ISI2. I plan to look for information related to food safety risk.	3.46	0.847
	ISI3. I will try my best to find out information related to food safety risk in the near future.	3.70	0.776

Notes: S.D. means standard deviation.

**Table 2:** Demographic characteristics of consumers (*N* = 774).

Characteristics	Variable Classification	Number	Ratio (%)
Gender	Female	425	54.9
	Male	349	45.1
Age	<35	507	65.5
	≥35	267	34.5
Education background	Below a bachelor’s degree	163	21.1
	Bachelor’s degree or higher	611	78.9
Average monthly earnings (in CNY)	≤5000	325	42.0
	>5000	325	42.0
	Missing	124	16.0

Notes: Exchange rate US$1 = 7.03 CNY (as of 11/30/2019).

**Table 3:** Reliability, validity, and confirmatory factor analysis results.

Latent Variables	Measurement Items	CITC	Cronbach’s α If Item Deleted	CR	AVE	KMO	Bartlett’s Test of Sphericity	Standard Factor Loadings
RP (Cronbach’s α = 0.93)	RP1	0.779	0.92	0.928	0.764	0.811	2802.031	0.79
	RP2	0.881	0.89					0.95
	RP3	0.893	0.88					0.96
	RP4	0.763	0.93					0.78
AR (Cronbach’s α = 0.92)	AR1	0.845	0.88	0.920	0.793	0.750	1730.367	0.89
	AR2	0.865	0.86					0.93
	AR3	0.801	0.91					0.85
II (Cronbach’s α = 0.89)	II1	0.802	0.81	0.884	0.718	0.741	1276.328	0.89
	II2	0.762	0.85					0.83
	II3	0.758	0.85					0.82
ISN (Cronbach’s α = 0.85)	ISN1	0.690	0.82	0.849	0.654	0.693	1053.206	0.78
	ISN2	0.795	0.71					0.88
	ISN3	0.670	0.83					0.76
RCB (Cronbach’s α = 0.85)	RCB1	0.759	0.75	0.852	0.658	0.720	1015.103	0.86
	RCB2	0.719	0.79					0.82
	RCB3	0.680	0.83					0.75
PIGC (Cronbach’s α = 0.90)	PIGC1	0.803	0.87	0.904	0.703	0.802	2088.629	0.88
	PIGC2	0.799	0.87					0.88
	PIGC3	0.784	0.88					0.81
	PIGC4	0.754	0.89					0.78
ISI (Cronbach’s α = 0.85)	ISI1	0.699	0.80	0.848	0.651	0.730	985.136	0.80
	ISI2	0.730	0.78					0.81
	ISI3	0.722	0.78					0.81

Notes: (1) Latent variables are RP = Risk Perception, AR = Affective Response, II = Information Insufficiency, ISN = Informational Subjective Norms, RCB = Relevant Channel Beliefs, PIGC = Perceived Information Gathering Capacity, ISI = Information-Seeking Intention. KMO = Kaiser–Meyer–Olkin measure of sampling adequacy. AVE = Average Variance Extracted (AVE). (2) Corrected Item-Total Correlation (CITC) values of each latent variable are higher than 0.5. The Cronbach’s alpha values of each latent variable are higher than 0.7, and Cronbach’s α if item deleted values of each measurement item are lower than the Cronbach’s alpha values of each latent variable, which means all measurement items should be kept. Composite Reliability (CR) values of the latent variable are higher than 0.5. It means the scale has high reliability. AVE values show that the latent variables have good convergence validity.

**Table 4:** The square root of AVE and correlation coefficients of latent variables.

Latent Variables	RP	AR	II	ISN	RCB	PIGC	ISI
RP	0.87						
AR	0.62	0.89					
II	0.26	0.12	0.85				
ISN	0.59	0.36	0.50	0.81			
RCB	0.30	0.19	0.25	0.50	0.81		
PIGC	0.19	0.12	0.21	0.42	0.45	0.84	
ISI	0.59	0.34	0.43	0.75	0.51	0.39	0.81

Notes: (1) Latent variables are RP = Risk Perception, AR = Affective Response, II = Information Insufficiency, ISN = Informational Subjective Norms, RCB = Relevant Channel Beliefs, PIGC = Perceived Information Gathering Capacity, ISI = Information-Seeking Intention. (2) The diagonal value at the top of each column (the square root of the AVE of each latent variable) is higher than other entries in the column (the correlation coefficients between one latent variable and other latent variables). It means that the latent variables have better discriminant validity.

**Table 5:** Fitting indices of the Structural Equation Model.

Classification	Fit Indices	Suggested Value	Actual Value	Fit Effect
Absolute Fit Measures	χ^2^/df	<5.00	6.37	Approx.
	RMSEA	<0.09	0.09	Accepted
	AGFI	>0.80	0.80	Accepted
	GFI	>0.90	0.85	Approx.
Incremental Fit Measures	CFI	>0.90	0.96	Accepted
	NFI	>0.95	0.96	Accepted
	NNFI	>0.95	0.96	Accepted
	IFI	>0.90	0.96	Accepted
Parsimonious Fit Measures	PNFI	>0.50	0.815	Accepted
	PGFI	>0.50	0.662	Accepted

Notes: RMSEA: Root Mean Square Error of Approximation. AGFI: Adjusted Goodness of Fit Index. GFI: Goodness of Fit Index. CFI: Comparative Fit Index. NFI: Normed Fit Index. NNFI: Non-normed Fit Index. IFI: Incremental Fit Index. PNFI: Parsimony Normed Fit Index. PGFI: Parsimony Goodness of Fit Index.

**Table 6:** Results from hypotheses tests between different latent variables in the Structural Equation Model.

Hypothesis	Standardized Coefficients	Direction	T-Value	Test Result
H1: RP→ISI	0.18	+	4.86	Support
H2: RP→AR	0.62	+	17.32	Support
H3: AR→II	0.07	−	−1.73	Support
H4: II→ISI	0.08	+	2.30	Support
H5: ISN→II	0.52	+	12.37	Support
H6: ISN→ISI	0.50	+	9.26	Support
H7: RCB→ISI	0.16	+	4.18	Support
H8: PIGC→ISI	0.06	+	1.60	Not support

Notes: Latent variables are RP = Risk Perception, AR = Affective Response, II = Information Insufficiency, ISN = Informational Subjective Norms, RCB = Relevant Channel Beliefs, PIGC = Perceived Information Gathering Capacity, ISI = Information-Seeking Intention.

**Table 7:** Multiple-group analysis results.

**Hypothesis**	**Gender**				**Age**			
	**Female**	***p*-Value**	**Male**	***p*-Value**	**<35**	***p*-Value**	**≥35**	***p*-Value**
H1	0.13 **	≤0.05	0.23 ***	≤0.01	0.19 ***	≤0.01	0.13 *	≤0.1
H2	0.63 ***	≤0.01	0.61 ***	≤0.01	0.65 ***	≤0.01	0.56 ***	≤0.01
H3	−0.02	>0.1	−0.13 **	≤0.05	−0.07	>0.1	−0.09	>0.1
H4	0.12 **	≤0.05	0.03	>0.1	0.09 **	≤0.05	0.01	>0.1
H5	0.40 ***	≤0.01	0.66 ***	≤0.01	0.54 ***	≤0.01	0.51 ***	≤0.01
H6	0.49 ***	≤0.01	0.51 ***	≤0.01	0.44 ***	≤0.01	0.63 ***	≤0.01
H7	0.11 **	≤0.05	0.21 ***	≤0.01	0.18 ***	≤0.01	0.13 **	≤0.05
H8	0.10 **	≤0.05	0.01	>0.1	0.14 ***	≤0.01	−0.03	>0.1
Hypothesis	Education Background				Average Monthly Earnings (in CNY)			
	Bachelor DegreeBlow	*p*-value	Bachelor Degree or Higher	*p*-value	≤5000	*p*-value	>5000	*p*-value
H1	0.11	>0.1	0.20 ***	≤0.01	0.10 *	≤0.1	0.26 ***	≤0.01
H2	0.63 ***	≤0.01	0.63 ***	≤0.01	0.61 ***	≤0.01	0.66 ***	≤0.01
H3	−0.20 ***	≤0.01	−0.02	>0.1	−0.09	>0.1	−0.07	>0.1
H4	−0.14	>0.1	0.13 ***	≤0.01	0.01	>0.1	0.12 **	≤0.05
H5	0.83 ***	≤0.01	0.43 ***	≤0.01	0.57 ***	≤0.01	0.26 ***	≤0.01
H6	0.69 ***	≤0.01	0.48 ***	≤0.01	0.59 ***	≤0.01	0.38 ***	≤0.01
H7	0.12	>0.1	0.16 ***	≤0.01	0.11 *	≤0.1	0.22 ***	≤0.01
H8	0.18 **	≤0.05	0.02	>0.1	0.23 ***	≤0.01	−0.03	>0.1

Notes: (1) ***, **, and * indicated that *p*-values are significant at 1%, 5%, and 10% levels respectively. (2) One hundred and twenty four respondents did not report average monthly earnings, so missing samples were excluded from multiple-group based on consumers’ characteristics of average monthly earnings.
